# Biological Variation in Rotational Thromboelastometry in Patients with Atrial Fibrillation Receiving Rivaroxaban

**DOI:** 10.3390/jcdd9070205

**Published:** 2022-06-29

**Authors:** Mojca Božič Mijovski, Jovan P. Antovic, Rickard E. Malmström, Alenka Mavri

**Affiliations:** 1 Department of Vascular Diseases, University Medical Centre Ljubljana, 1000 Ljubljana, Slovenia; alenka.mavri@kclj.si; 2 Faculty of Pharmacy, University of Ljubljana, 1000 Ljubljana, Slovenia; 3 Department of Coagulation Research, Institute for Molecular Medicine and Surgery, Karolinska Institutet, 17177 Stockholm, Sweden; jovan.antovic@ki.se; 4 Department of Clinical Chemistry, Karolinska University Hospital, 17177 Stockholm, Sweden; 5 Department of Medicine Solna, Karolinska Institutet, Karolinska University Hospital, 17177 Stockholm, Sweden; rickard.malmstrom@ki.se; 6 Clinical Pharmacology, Karolinska University Hospital, 17177 Stockholm, Sweden

**Keywords:** rivaroxaban, rotational thromboelastometry, biological variation, atrial fibrillation

## Abstract

Rotational thromboelastometry (ROTEM) is a viscoelastic hemostasis test used primarily in the management of bleeding after trauma or in cardiac surgery. To allow safe and valid clinical interpretation of test results, objective specifications for analytical performance are needed, which are generally based on biological variation within (CV_I_) and between (CV_G_) individuals. The aim of this study was to evaluate biological variation in ROTEM in patients receiving rivaroxaban. Sixty patients with atrial fibrillation on stable rivaroxaban therapy were included, from whom blood was collected on six occasions: three times at trough and three at peak rivaroxaban concentrations. ROTEM^®^ Extem and LowTF were measured as well as rivaroxaban concentration, PT, APTT, and anti-Xa. Within- (CV_I_) and between-subject (CV_G_) biological estimates were calculated. Knowledge of these biological variation components will help to establish the appropriate objective analytical performance specifications for ROTEM analysis.

## 1. Introduction

Rotational thromboelastometry (ROTEM) is a viscoelastic hemostasis test used primarily in the management of bleeding after trauma or in cardiac surgery. ROTEM can detect the contribution of plasma and cellular elements to hemostasis. Results of the meta-analysis showed a benefit of using a ROTEM-guided transfusion [1]. Its turnaround time is usually shorter compared to screening coagulation tests and may be more accessible than specific coagulation tests, such as anti-Xa [2]. Anti-Xa is used to measure the anticoagulant effect of heparin and its derivatives and of direct oral factor Xa inhibitors such as rivaroxaban [3]. However, anti-Xa is rarely available 24/7, especially when used for direct oral factor Xa inhibitors, which require specific calibrators and controls. Therefore, other more readily available methods would be of great value, given the widespread use of rivaroxaban and the critical importance of assessing its plasma levels in situations such as bleeding or emergency surgery [4]. Several studies have examined changes in ROTEM results obtained in plasma samples spiked with rivaroxaban [5,6], in samples from healthy subjects taking rivaroxaban [7,8], and in patients receiving rivaroxaban therapy [9,10,11,12]. In ROTEM, clotting can be induced by tissue factor (Extem) or with a contact activator (Intem). Additional tests are also available (Fibtem, Aptem, Heptem), which are compared with Extem or Intem. Several studies showed that Extem was the most sensitive to rivaroxaban [7,8,9,13]. A variant of the EXTEM assay with lower tissue factor concentration (LowTF) was also described and showed increased sensitivity and specificity to rivaroxaban compared to the classic Extem [14,15,16].

To allow the safe and valid clinical interpretation of test results, including ROTEM, objective specifications for analytical performance are needed. These are generally based on biological variation within (CV_I_) and between (CV_G_) individuals. However, information on biological variation in hemostasis variables is still very limited [17]. Therefore, the aim of this study was to evaluate the biological variation in ROTEM Extem and LowTF, as well as prothrombin time (PT), activated partial thromboplastin time (APTT) and anti-Xa in patients receiving rivaroxaban.

## 2. Patients and Methods

Sixty patients with atrial fibrillation were selected from the Anticoagulation Clinic (University Medical Centre Ljubljana) registry [18]. Thirty patients were on rivaroxaban 20 mg daily and 30 patients on rivaroxaban 15 mg daily. The lower-dose rivaroxaban was prescribed to patients with moderate renal impairment (CrCl 30–50 mL/min), high bleeding risk, or previous major bleeding, at the discretion of the treating physician. Demographic data, thromboembolic, and hemorrhagic risks according to the CHADS2 and HAS-BLED scoring system were recorded. Renal function was estimated using the Cockcroft–Gault equation. Two patients (out of sixty) were treated with antiplatelet agents: one with acetylsalicylic acid and one with clopidogrel. Three patients received amiodarone, which is considered a mild glycoprotein P inhibitor. None of the patients received potent inhibitors of glycoprotein P or cytochrome P450. Patients were treated for hypertension, diabetes, and heart failure as needed (Table 1). One patient received methylprednisolone. From all patients, three trough (trough 1, 2, and 3) and three peak (peak 1, 2, and 3) blood samples were collected for determination of rivaroxaban drug concentration and ROTEM with an interval of 6–8 weeks apart. Two patients missed one appointment each; therefore, 358 blood samples were available for analysis. The trough concentration samples were collected 24 ± 1 h after the previous rivaroxaban dose, and the peak concentration samples were collected 124 ± 8 min after intake of rivaroxaban with food.

All patients signed an informed consent form agreeing to participate in the study. The study was approved by the Medical Ethical Committee of the Slovenian Ministry of Health.

Blood was collected in two 4.5 mL vacuum tubes containing 0.11 mol/L sodium citrate (Becton Dickinson, Vacutainer System Europe, Heidelberg, Germany). One tube was used for ROTEM^®^ Extem and LowTF performed on ROTEM^®^ delta analyzer (all Tem Innovations GmbH, Germany) within one hour of blood collection, according to the manufacturer’s instructions (Extem) or with the diluted tissue factor (Dade Innovin^®^, Siemens Healthcare, Marburg, Germany) as described by Adelmann et al. [14]. The following parameters were recorded: CT, clot formation time (CFT), and maximum clot firmness (MCF). The second blood tube was centrifuged for 30 min at 2000× g and 4 °C. In plasma, rivaroxaban concentration was measured with liquid chromatography and tandem mass spectrometry (LC-MS/MS) as described previously [19]. PT, APTT, and anti-Xa were measured with Thromborel S, Pathromtin SL and Innovance Heparin, respectively (all Siemens Healthcare, Germany), on a CS-2500 coagulation analyzer (Sysmex, Kobe, Japan). Anti-Xa was calibrated with STA–Rivaroxaban Calibrator (Diagnostica Stago, Asnières sur Seine, France).

### Statistical Analysis

Data are presented as medians with first to third quartile and correlation with Spearman’s coefficient r. CV_I_ and CV_G_ estimates were derived from logarithmically transformed data after excluding three samples with the rivaroxaban concentration below the limit of quantification obtained with the LC-MS/MS [20]. Estimates of standard deviations (σ) were calculated from logarithmically transformed data as σ_I_ and σ_G_. The σ was then retransformed into CV_I_ and CV_G_ using the following formula [20]:

$$CVln=\sqrt{\left(\exp\;\sigma^{2}-1\right)}\;\times \;100$$

## 3. Results

Patient characteristics are listed in Table 1. Patients receiving the lower dose of rivaroxaban (R15) were significantly older, more often female, and had significantly lower body weight and CrCl and higher CHADS2 and HAS-BLED scores than patients receiving 20 mg of rivaroxaban daily (R20). The two patient groups did not differ in the prevalence of arterial hypertension, diabetes mellitus, heart failure, ischemic heart disease, prior stroke, or peripheral artery disease.

Rivaroxaban concentrations and all coagulation parameters were similar in the R20 and R15 patients. In addition, none of the parameters that differed between the R20 and R15 groups (age, sex, body weight, creatinine, CrCl, CHADS_2_, and HAS-BLED scores) correlated with rivaroxaban concentration or ROTEM parameters, so the data were pooled for the calculation of biological variations. The results of all laboratory tests at the trough and peak concentrations of rivaroxaban are shown in Table 2. There was no difference between the three trough and three peak levels for any of the variables measured. With the exception of ROTEM Extem MCF and LowTF MCF, all measured variables were higher in the peak than in the trough. Antiplatelet agents, amiodarone, and methylprednisolone had no effect on rivaroxaban concentrations or on coagulation tests.

**Figure 1:**
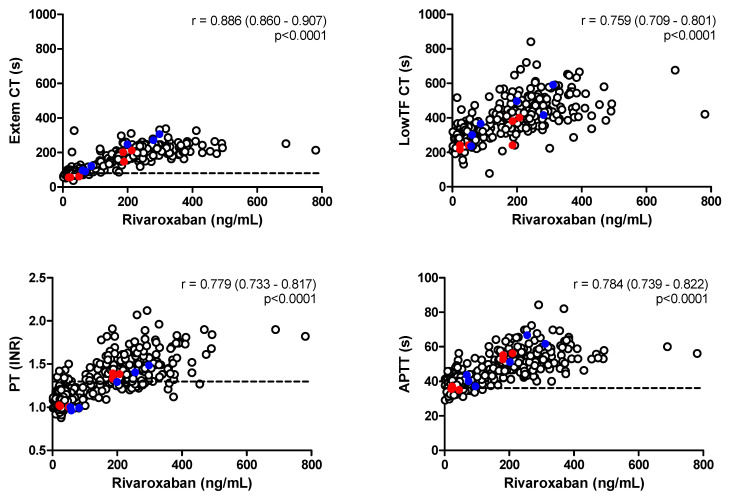
Correlation between rivaroxaban concentration and ROTEM Extem CT, ROTEM LowTF, PT, and APTT. Pearson’s correlation coefficient is given with the 95% confidence interval and statistical significance. Dotted lines represent the upper reference value. Results from patients receiving antiplatelet therapy are shown in color (red for acetylsalicylic acid and blue for clopidogrel).

ROTEM Extem CT correlated significantly with rivaroxaban concentration (Figure 1). A much lower but still significant correlation was observed for Extem CFT (Spearman’s r = 0.251, *p* <0.0001), and no correlation for MCF. Similarly, the correlation between ROTEM LowTF CT and rivaroxaban concentration was highly significant (Figure 1), as was the correlation between LowTF CFT and rivaroxaban concentration (r = 0.516, *p* < 0.0001), and there was a significant negative correlation between LowTF MCF and rivaroxaban concentration (r = −0.194, *p* < 0.001). Both PT and APTT showed a similar correlation to rivaroxaban concentration as ROTEM Extem CT and LowTF CT (Figure 1).

Intra- (CV_I_) and interindividual (CV_G_) biological variations were calculated separately for trough and peak values for all the measured variables and are shown in Table 3. As expected, interindividual variations were higher than intraindividual variations. The highest CV_G_ was observed for anti-Xa (87.7 %) and rivaroxaban concentration (80.0 %). The lowest CVs were observed for ROTEM Extem MCF, which did not correlate with the rivaroxaban concentration.

## 4. Discussion

The aim of the present study was to evaluate the biological intra- (CV_I_) and inter-individual (CV_G_) variation in ROTEM Extem and LowTF Extem in patients on stable rivaroxaban therapy. Biological variation in standard coagulation tests PT, APTT, and anti-Xa was also evaluated.

There is no defined therapeutic range for rivaroxaban, but the rivaroxaban concentrations detected at both the trough and the peak in our study were similar to those observed in other studies in which the reference method (liquid chromatography with tandem mass spectrometry) was used [21,22,23]. A significant positive correlation between rivaroxaban concentration and Extem CT in patients receiving rivaroxaban has been reported by others [9,13], but in our study, this correlation was even higher, probably because of a much higher number of samples (n = 358). Reports on the correlation between rivaroxaban concentration and Extem CFT are varied. In one study, a significant positive correlation was found between rivaroxaban and Extem CFT in 10 healthy volunteers who took a single dose of 10 mg rivaroxaban [7]. Another study of 20 healthy volunteers found a negative correlation [8], and three studies (two ex vivo and one in vitro) reported no correlation [5,9,13]. In none of these studies was the number of samples more than 20 compared with 358 samples in our study, which confirmed a small but highly significant positive correlation between rivaroxaban and Extem CFT. No correlation between rivaroxaban and MCF was observed in any of the studies, including ours. We found a significant positive correlation between rivaroxaban and LowTF CT and LowTF CFT, similar to the study by Adelmann et al. [14]. We also found a significant negative correlation between rivaroxaban and LowTF MCF. This result cannot be directly compared with the results of Adelmann et al. [14] because they did not report the MCF value, but they found a lower clot firmness at 10 min (A10), so we can assume that the MCF (read from the ROTEM curve a few minutes after A10) would also be decreased. The correlations between rivaroxaban and the coagulation tests PT, APTT, and anti-Xa found in this study are consistent with those reported by others [24].

Six patients in our study received medications that could affect rivaroxaban blood concentrations or ROTEM results: one patient was treated with acetylsalicylic acid, one patient was treated with clopidogrel, three patients received amiodarone, and one patient received methylprednisolone. We did not detect any effects of these medications on rivaroxaban concentrations or ROTEM results, but because of the small number of patients, we cannot assume that there are no subtle effects. However, this should be confirmed in a larger group of patients.

We examined CV_I_ and CV_G_ in patients with atrial fibrillation on stable rivaroxaban therapy and found that the highest variation in rivaroxaban concentration occurred between individuals (CV_G_) at the trough (80 %), whereas CV_G_ was much lower at the peak. CV_I_ was also higher at the trough concentration than at the peak concentration of rivaroxaban. Comparable variations were observed when rivaroxaban concentrations were determined indirectly via anti-Xa. This is not surprising, as an anti-Xa has replaced cumbersome liquid chromatography with tandem mass spectrometry in routine clinical practice [24].

The ROTEM parameters with the highest CV_G_ were Extem CT, Extem CFT, LowTF CT, and LowTF CFT for both trough and peak. CV_I_ and CV_G_ for Extem and LowTF MCF were less than 10 %. Data on biological variation in ROTEM are virtually nonexistent. We found one study reporting CV_I_ and CV_G_ in ten healthy men using the ROTEM Natem test, which initiates clotting with recalcification of blood samples [25]. This test was replaced by Intem, which also uses a contact activator (elagic acid) and partial thromboplastin. Compared with this study, we found higher CV_G_ and lower CV_I_ for CT (both Extem and LowTF). In addition to the analytical differences between these assays, the higher CV_G_ may be due to a high variation in rivaroxaban concentration, whereas the lower CV_I_ may be due to a larger sample number; however, further studies are needed to confirm these results.

For PT and APTT, variability depends on the sensitivity of the reagent used [25]. In our study, thromboplastin with low sensitivity to rivaroxaban was used. Therefore, it is not surprising that CV_I_ at trough concentration was similar to that in healthy subjects [24] but was higher at peak concentration. CV_I_ and CV_G_ for APTT at trough and at peak were higher than in healthy subjects because we used a sensitive reagent [26,27].

The limitations of our study were that only patients on rivaroxaban therapy were included and that the assays were not performed in duplicate to obtain the corresponding analytical variation.

The strengths of the study are the large number of patients included and the rigorous preanalytical protocols (all samples were analyzed within one hour of blood collection), as well as the appropriate statistical methods used to obtain reliable biological variation data.

## 5. Conclusions

In conclusion, this study provides insights into the biological variability of ROTEM Extem and LowTF in patients on stable rivaroxaban therapy. The knowledge of these biological variation components will help to establish the appropriate objective analytical performance specifications and calculate the reference change value to decide whether there is a significant difference between the two test results from the same individual. This is particularly important in bleeding patients in whom transfusion or the use of a rivaroxaban neutralizing agent may be considered.

## Figures and Tables

**Table 1:** Characteristics of the patients on rivaroxaban 20 mg (R20) or 15 mg (R15) daily. Average ± SD or number of cases is given with the percentage.

	R20	R15	*p*
Age (years)	71 ± 6	76 ± 6	<0.01
Sex (women/men)	9/21	19/11	0.02
Body weight (kg)	90 ± 17	79 ± 16	0.01
Creatinine (μmol/L)	76 ± 14	98 ± 20	<0.001
CrCl (mL/min)	99 ± 32	60 ± 26	<0.001
Arterial hypertension (*N*, %)	26 (87)	28 (93)	NS
Diabetes mellitus (*N*, %)	4 (13)	9 (30)	NS
Heart failure (*N*, %)	6 (20)	8 (27)	NS
Ischemic heart disease (*N*, %)	6 (20)	6 (20)	NS
Previous stroke or systemic embolism (*N,* %)	3 (10)	5 (17)	NS
Peripheral artery disease (*N*, %)	1 (3)	1 (3)	NS
CHADS_2_ score	1.8 ± 1.3	2.5 ± 1.2	0.01
HAS-BLED score	0.9 ± 0.7	1.2 ± 0.5	0.02

**Table 2:** Rivaroxaban concentration, ROTEM, PT, and APTT results at trough and at peak. Medians with first to third quartiles are shown.

Measurand	Trough 1	Trough 2	Trough 3	Peak 1	Peak 2	Peak 3
Rivaroxaban (ng/mL)	34 (19–52)	33 (16–50)	36 (24–50)	244 (193–308)	240 (190–286)	244 (188–312)
ROTEM Extem						
CT (s)	85 (74–95)	81 (73–93)	83 (73–97)	205 (176–232)	189 (166–220)	202 (179–242)
CFT (s)	69 (58–80)	72 (60–83)	68 (58–81)	73 (67–89)	77 (70–88)	74 (66–81)
MCF (mm)	66 (64–70)	66 (62–70)	66 (63–69)	66 (63–69)	65 (63–68)	65 (62–68)
ROTEM LowTF						
CT (s)	276 (253–311)	262 (239–286)	290 (260–319)	452 (399–526)	404 (363–465)	448 (410–539)
CFT (s)	101 (89–118)	95 (86–121)	99 (89–118)	139 (113–164)	128 (113–157)	134 (119–168)
MCF (mm)	63 (61–67)	63 (61–67)	63 (60–66)	62 (58–65)	62 (58–65)	61 (57–64)
PT (INR)	1.1 (1.0–1.1)	1.1 (1.0–1.1)	1.1 (1.0–1.1)	1.5 (1.3–1.6)	1.4 (1.3–1.6)	1.4 (1.3–1.6)
APTT (s)	38.1 (34.8–40.7)	37.7 (35.2–40.5)	37.6 (35.3–39.8)	52.4 (46.9–59.0)	51.5 (47.0–58.7)	51.5 (46.9–55.9)
Anti-Xa (ng/mL)	32 (19–46)	29 (17–44)	31 (19–44)	237 (208–358)	240 (220–317)	243 (217–348)

**Table 3:** Within- (CV_I_) and between-subject (CV_G_) biological estimates for rivaroxaban concentration, ROTEM Extem, ROTEM LowTF, PT, APTT, and anti-Xa.

Measurand	CV_I_ Trough (%)	CV_I_ Peak (%)	CV_G_ Trough (%)	CV_G_ Peak (%)
Rivaroxaban (ng/mL)	35.4	19.9	80.0	37.1
ROTEM Extem				
CT (s)	12.7	11.8	23.8	21.2
CFT (s)	10.7	13.2	22.3	18.7
MCF (mm)	2.5	3.1	6.5	6.3
ROTEM LowTF				
CT (s)	13.2	19.0	17.8	24.5
CFT (s)	16.5	23.0	25.0	29.7
MCF (mm)	3.1	4.9	7.3	9.3
PT (INR)	2.3	5.8	8.9	15.2
APTT (s)	4.2	5.6	11.2	14.1
Anti-Xa (ng/mL)	34.7	16.7	86.7	35.4

## Data Availability

Raw is not publicly available due to patient confidentiality.

## References

[1] Wikkelsø A., Wetterslev J., Møller A.M., Afshari A. (2017). Thromboelastography (TEG) or rotational thromboelastometry (ROTEM) to monitor haemostatic treatment in bleeding patients: A systematic review with meta-analysis and trial sequential analysis. Anaesthesia.

[2] Korpallová B., Samoš M., Bolek T., Kühnelová L., Škorňová I., Kubisz P., Staško J., Mokáň M. (2021). ROTEM Testing for Direct Oral Anticoagulants. Semin. Thromb. Hemost..

[3] Mijovski M.B. (2019). Advances in monitoring anticoagulant therapy. Adv. Clin. Chem..

[4] Cuker A., Siegal D. (2015). Monitoring and reversal of direct oral anticoagulants. Hematol. Am. Soc. Hematol. Educ. Program..

[5] Eller T., Busse J., Dittrich M., Flieder T., Alban S., Knabbe C., Birschmann I. (2014). Dabigatran, rivaroxaban, apixaban, argatroban and fondaparinux and their effects on coagulation POC and platelet function tests. Clin. Chem. Lab. Med..

[6] Seyve L., Richarme C., Polack B., Marlu R. (2017). Impact of four direct oral anticoagulants on rotational thromboelastometry (ROTEM). Int. J. Lab. Hematol..

[7] Casutt M., Konrad C., Schuepfer G. (2012). Effect of rivaroxaban on blood coagulation using the viscoelastic coagulation test ROTEM™. Anaesthesist.

[8] Fontana P., Alberio L., Angelillo-Scherrer A., Asmis L.M., Korte W., Mendez A., Schmid P., Stricker H., Studt J.-D., Tsakiris D.A. (2017). Impact of rivaroxaban on point-of-care assays. Thromb. Res..

[9] Chojnowski K., Górski T., Robak M., Treliński J. (2015). Effects of Rivaroxaban Therapy on ROTEM Coagulation Parameters in Patients with Venous Thromboembolism. Adv. Clin. Exp. Med..

[10] Pailleret C., Jourdi G., Siguret V., Gouin-Thibault I., Gandrille S., Stepanian A., Curis E., Golmard J.L., Gaussem P., Le Bonniec B. (2019). Modified ROTEM for the detection of rivaroxaban and apixaban anticoagulant activity in whole blood: A diagnostic test study. Eur. J. Anaesthesiol..

[11] Tsantes A.E., Kyriakou E., Ikonomidis I., Katogiannis K., Papadakis I., Douramani P., Kopterides P., Kapsimali V., Lekakis J., Tsangaris I. (2016). Comparative Assessment of the Anticoagulant Activity of Rivaroxaban and Dabigatran in Patients with Nonvalvular Atrial Fibrillation: A Noninterventional Study. Medicine.

[12] Vedovati M.C., Mosconi M.G., Isidori F., Agnelli G., Becattini C. (2020). Global thromboelastometry in patients receiving direct oral anticoagulants: The RO-DOA study. J. Thromb. Thrombolysis.

[13] Klages M., Raimann F.J., Philipp A.-L., Lindhoff-Last E., Zacharowski K., Mutlak H. (2021). Direct oral anticoagulants in point-of-care monitoring: An ex-vivo study. Minerva Anestesiol..

[14] Adelmann D., Wiegele M., Wohlgemuth R.K., Koch S., Frantal S., Quehenberger P., Scharbert G., Kozek-Langenecker S., Schaden E. (2014). Measuring the activity of apixaban and rivaroxaban with rotational thrombelastometry. Thromb. Res..

[15] Schafer S.T., Otto A.C., Acevedo A.C., Gorlinger K., Massberg S., Kammerer T., Groene P. (2021). Point-of-care detection and differentiation of anticoagulant therapy—Development of thromboelastometry-guided decision-making support algorithms. Thromb. J..

[16] Schäfer S.T., Wiederkehr T., Kammerer T., Acevedo A.-C., Feil K., Kellert L., Görlinger K., Hinske L.C., Groene P. (2020). Real-time detection and differentiation of direct oral anticoagulants (rivaroxaban and dabigatran) using modified thromboelastometric reagents. Thromb. Res..

[17] https://biologicalvariation.eu.

[18] Miklič M., Mavri A., Vene N., Söderblom L., Božič-Mijovski M., Pohanka A., Antovic J., Malmström R.E. (2019). Intra- and inter- individual rivaroxaban concentrations and potential bleeding risk in patients with atrial fibrillation. Eur. J. Clin. Pharmacol..

[19] Al-Aieshy F., Malmström R.E., Antovic J., Pohanka A., Rönquist-Nii Y., Berndtsson M., Al-Khalili F., Skeppholm M. (2016). Clinical evaluation of laboratory methods to monitor exposure of rivaroxaban at trough and peak in patients with atrial fibrillation. Eur. J. Clin. Pharmacol..

[20] Røraas T., Støve B., Petersen P.H., Sandberg S. (2016). Biological Variation: The Effect of Different Distributions on Estimated Within-Person Variation and Reference Change Values. Clin. Chem..

[21] Bardy G., Fischer F., Appert A., Baldin B., Stève M., Spreux A., Lavrut T., Drici M.-D. (2015). Is anti-factor Xa chromogenic assay for Rivaroxaban appropriate in clinical practice? Advantages and comparative drawbacks. Thromb. Res..

[22] Bento Matos Derogis P., Rentas Sanches L., De Aranda V.F., Colombini M.P., Mangueira C., Katz M., Caschera Leme Faulhaber A., Mendes C.E.A., Ferreira C.E.D.S., França C.N. (2017). Determination of rivaroxaban in patient’s plasma samples by anti-Xa chromogenic test associated to High Performance Liquid Chromatography tandem Mass Spectrometry (HPLC-MS/MS). PLoS ONE.

[23] Cini M., Legnani C., Padrini R., Cosmi B., Dellanoce C., De Rosa G., Marcucci R., Pengo V., Poli D., Testa S. (2020). DOAC plasma levels measured by chromogenic anti-Xa assays and HPLC-UV in apixaban- and rivaroxaban-treated patients from the START-Register. Int. J. Lab. Hematol..

[24] Baglin T., Hillarp A., Tripodi A., Elalamy I., Buller H., Ageno W. (2013). Measuring Oral Direct Inhibitors (ODIs) of thrombin and factor Xa: A recommendation from the Subcommittee on Control of Anticoagulation of the Scientific and Standardisation Committee of the International Society on Thrombosis and Haemostasis. J. Thromb. Haemost..

[25] Jilma-Stohlawetz P., Fritsche-Polanz S., Quehenberger P., Schoergenhofer C., Bartko J., Ristl R., Jilma B. (2017). Evaluation of between-, within- and day-to-day variation of coagulation measured by rotational thrombelastometry (ROTEM). Scand. J. Clin. Lab. Investig..

[26] Falay M., Senes M., Korkmaz S., Turhan T., Okay M., Öztürk B.A., Yücel D., Ozet G. (2018). Biological variation estimates of prothrombin time, activated partial thromboplastin time, and fibrinogen in 28 healthy individuals. Int. J. Lab. Hematol..

[27] Henskens Y.M.C., Gulpen A.J.W., Van Oerle R., Wetzels R., Verhezen P., Spronk H., Schalla S., Crijns H.J., Cate H.T., Cate-Hoek A.T. (2018). Detecting clinically relevant rivaroxaban or dabigatran levels by routine coagulation tests or thromboelastography in a cohort of patients with atrial fibrillation. Thromb. J..

